# Supplementary material for: An Asymptomatic, Ectopic Mass as a Presentation of Adrenocortical Carcinoma Due to a Novel Germline TP53 p.Phe338Leu Tetramerisation Domain Variant
Source: Children (Basel). 2023 Nov 7;10(11):1793. doi: 10.3390/children10111793 (PMC10670401; doi:10.3390/children10111793)

Molecular karyotype

arr[GRCh37] 1p36.33p35.1(11828_33795701)x3[0.2],1q44(247365848_249213976)x1[0.15~0.2],(3)x3[0.4],
(4p)x3,4p15.33p11(15131588_49051765)x3 hmz,(4q)x1[0.45],(7)x3[0.4],(8)x2 hmz,9p21.1q21.11(33025604_71021717)x3[0.3~0.5],
9q22.2q31.1(93219948_102845908)x3[0.4],9q31.1q34.3(102861967_141134447)x3~4,9q31.1q31.2(103910778_110112190)x3~4 hmz,
9q31.3q34.3(111525039_141011581)x3~4 hmz,(11)x2 hmz,(12)x2~3,12p13.33p13.31(192220_6277311)x2~3 hmz,(13)x3[0.65],
15q11.1q24.3(20636006_77712455)x3[0.6],15q24.3q26.3(77739599_102519341)x4[0.4],17p13.3p12(5343_10944407)x1,
17p13.1p11.1(10565963_22217680)x2 hmz,17q11.1q25.3(25343371_81041938)x2 hmz,18q21.33q23(61583609_78005819)x2~<2,
(19,20)x3[0.6],21q11.2q21.3(14410158_28217699)amp hmz,21q21.3q22.12(28231042_36811656)x3 hmz[0.4]

Karyoview

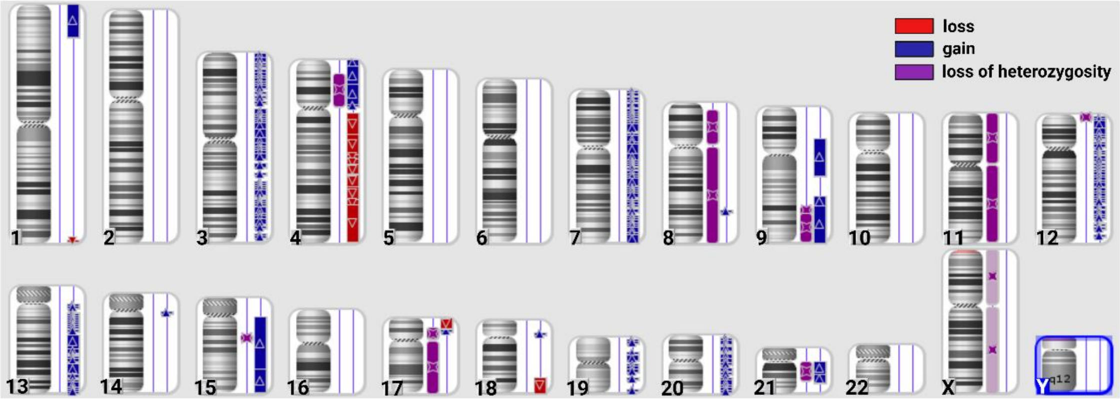

Whole genome view

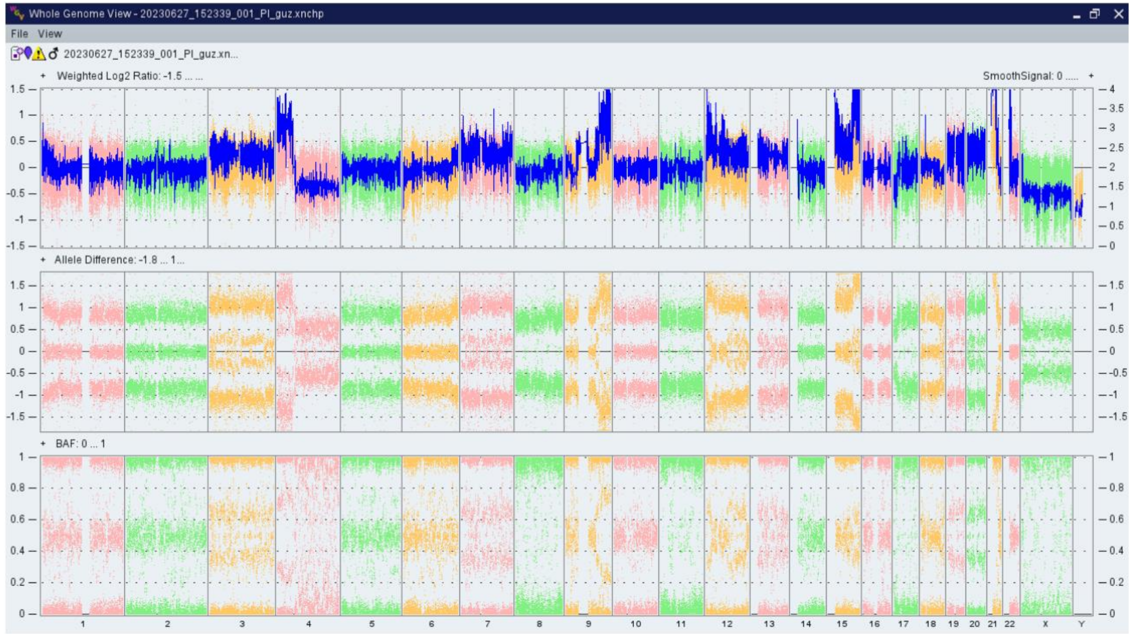

Supplement: Supplementary file 1 [file children-10-01793-s001.zip › children-2664936-supplementary/Supplementary Figure S1.pdf]
