# Supplementary material for: An Asymptomatic, Ectopic Mass as a Presentation of Adrenocortical Carcinoma Due to a Novel Germline TP53 p.Phe338Leu Tetramerisation Domain Variant
Source: Children (Basel). 2023 Nov 7;10(11):1793. doi: 10.3390/children10111793 (PMC10670401; doi:10.3390/children10111793)
Supplement: Supplementary file 1 [file children-10-01793-s001.zip › children-2664936-supplementary/Supplementary Table S1.pdf]

Variant	TP53(NM_000546.6):c.1012T>C, p.Phe338Leu	
**Frequency**		
**gnomAD Genomes**	not found	
**gnomAD exomes**	not found	
**Conservation**		
**Scores**	**Value**	
**PhastCons100way**	0.897	
**PhyloP100way**	8.183	
**Pathogenicity Scores**		
**Meta scores**		
**Engine**	**Calibrated Prediction**	**Score**
BayesDel addAF	Pathogenic Moderate	0.2279
MetaLR	Pathogenic Supporting	0.8532
MetaRNN	Pathogenic Supporting	0.7701
MetaSVM	Pathogenic Supporting	0.8849
BayesDel noAF	Uncertain	0.08959
REVEL	Uncertain	0.681
**Individual Predictions**		
DEOGEN2	Pathogenic Moderate	0.9628
EVE	Pathogenic Supporting	0.6595
FATHMM-MKL	Pathogenic Supporting	0.9935
FATHMM-XF	Pathogenic Supporting	0.9229
LIST-S2	Pathogenic Supporting	0.9725
M-CAP	Pathogenic Supporting	0.4196
MutPred	Pathogenic Supporting	0.706
LRT	Benign Supporting	0.005234
BLOSUM	Uncertain	0
CADD	Uncertain	25.3999
DANN	Uncertain	0.9961
EIGEN	Uncertain	0.3318
FATHMM	Uncertain	-3.47
Mutation assessor	Uncertain	2.77
MutationTaster	Uncertain	0.9985
MVP	Uncertain	0.9348
Polyphen2 HDIV	Uncertain	0.787
Polyphen2 HVAR	Uncertain	0.595
PrimateAI	Uncertain	0.5514
PROVEAN	Uncertain	-3.77
SIFT	Uncertain	0.002
SIFT4G	Uncertain	0.005

**Version**
version 14-Apr-2021
version 13-Apr-2021
**Version**
dbNSFP version 4.3
dbNSFP version 4.3
dbNSFP version 4.3
dbNSFP version 4.3
dbNSFP version 4.3
dbNSFP version 4.3
dbNSFP version 4.3
version 07-Jun-2022
dbNSFP version 4.3
dbNSFP version 4.3
dbNSFP version 4.3
dbNSFP version 4.3
dbNSFP version 4.3
dbNSFP version 4.3
version BLOSUM100
version 1.6
dbNSFP version 4.3
dbNSFP version 4.3
dbNSFP version 4.3
dbNSFP version 4.3
dbNSFP version 4.3
version 4.3
version 4.3
dbNSFP version 4.3
dbNSFP version 4.3
dbNSFP version 4.3
dbNSFP version 4.3
